# Long intergenic noncoding RNA 00665 promotes proliferation and inhibits apoptosis in colorectal cancer by regulating miR-126-5p

**DOI:** 10.18632/aging.202874

**Published:** 2021-04-20

**Authors:** Chang-Liang Wu, Ti-Dong Shan, Yue Han, Yan Kong, Yuan-Bo Li, Xin-Gang Peng, Liang Shang, Pei-Ge Wang, Le-Ping Li

**Affiliations:** 1 Department of Gastrointestinal Surgery, Shandong Provincial Hospital, Cheeloo College of Medicine, Shandong University, Jinan 250021, Shandong, People’s Republic of China; 2 Department of Emergency Surgery, The Affiliated Hospital of Qingdao University, Qingdao University, Qingdao 262000, Shandong, People’s Republic of China; 3 Department of Gastroenterology, The Affiliated Hospital of Qingdao University, Qingdao University, Qingdao 262000, Shandong, People’s Republic of China; 4 Department of PET-CT, The Affiliated Hospital of Qingdao University, Qingdao University, Qingdao 262000, Shandong, People’s Republic of China

**Keywords:** LINC00665, miR-126-5p, colorectal cancer, proliferation, apoptosis

## Abstract

Long intergenic noncoding RNAs (lincRNAs) regulate a series of biological processes, and their anomalous expression plays critical roles in the progression of multiple malignancies, including colorectal cancer (CRC). Although many studies have reported the oncogenic function of LINC00665 in multiple cancers, few studies have explored its role in CRC. The aim of this study was to assess the effect of LINC00665 on the malignant behaviors of CRC and explore the underlying regulatory mechanism of LINC00665. LINC00665 was significantly upregulated in CRC. A loss-of-function assay revealed that LINC00665 downregulation inhibited the proliferation and promoted the apoptosis of CRC cells, which was mediated by cyclin D1, CDK4, caspase-9 and caspase-3. Through mechanistic exploration, we found that miR-126-5p directly bound to LINC00665. Moreover, LINC00665 and miR-126-5p both regulated PAK2 and FZD3 expression. Mechanistically, miR-126-5p was predicted and further verified as a target of both PAK2 and FZD3. These findings demonstrate that LINC00665 might play an important pro-proliferative and antiapoptotic role in CRC and might be a potential biomarker and a new therapeutic target for CRC.

## INTRODUCTION

Colorectal cancer (CRC) is currently considered to be the disease with the third highest mortality rate and tumor-related mortality worldwide [1]. Early diagnosis of CRC can enable a better prognosis through medical treatment options. However, the diagnosis and treatment of advanced or metastatic CRC is still very scarce. Although targeted therapies have been successfully used to treat certain types of tumors, their performance in the treatment of CRC is not satisfactory, and for patients with advanced disease, their survival benefit is relatively small [2, 3]. Therefore, the timely identification of novel biomarkers and further evaluation of their detection value are crucial for the treatment of CRC.

Noncoding RNAs (ncRNAs) participate in gene regulation to further influence the biological behavior of cells [4]. Long noncoding RNAs (lncRNAs) are a type of RNA with a length greater than 200 nucleotides and without protein-coding function [5]. Long intergenic noncoding RNAs (lincRNAs) are a subspecies of lncRNAs [6]. LncRNAs derived from intergenic regions between two protein-coding genes are classified as lincRNAs [7]. A previous study found that lincRNAs participate in various biological behaviors of various cancers and emphasized their significance as biomarkers or therapeutic targets [4]. The mechanisms of action of lincRNAs in the context of CRC are not fully defined; therefore, exploring the roles of lincRNAs in CRC could develop a new direction of diagnosis and treatment. MicroRNAs (miRNAs) are considered short ncRNA fragments and negatively regulate gene transcript function. Studies have proven that lncRNAs regulate mRNA expression through their miRNA sponge, named the competitive endogenous RNA (ceRNA) network, and this function is closely related to tumor development [8]. Thus, we proposed to investigate the ceRNA network in CRC in this study.

The Wnt/β-catenin pathway seems to be a main factor during carcinogenesis among different cancers. Studies have shown that 10 Frizzled receptors (FZD1–10) are distributed in the highly conserved WNT network, which guides the dynamic balance of cell metabolism [9–11]. FZDs can transmit extracellular signals to the output of a variety of transcription programs, which determine cellular outcomes in pathological and physiological processes [12]. Studies have indicated that downregulation of the FZD3 receptor inhibits proliferation in human melanoma through the WNT signaling pathway [13]. p21 activated kinase 2 (PAK2) belongs to the PAK family, which acts as a downstream substrate of GTPases Rac and CDC42 [14, 15]. The PAK family (PAK 1-6) participates in the biological behavior of different tumor types, such as proliferation and apoptosis [16, 17]. Among them, PAK2 may be related to the progression and prognosis of tumors and has the potential to become a cancer therapy target [18–20]. Accordingly, FZD3 and PAK2 and their roles in tumors need to be considered.

The present study was designed to provide new ideas and perspectives for the role of LINC00665 and determine its underlying mechanism in CRC. In this research, the expression of LINC00665 in CRC tissues and cell lines was detected. Additionally, we also assessed the relationship between LINC00665 expression and miR-126-5p. Overall, we determined that LINC00665 acted as an oncogene by modulating miR-126-5p, thereby aggravating tumorigenesis, demonstrating that LINC00665 might be a valuable and promising therapeutic target for CRC.

## RESULTS

### LINC00665 expression was overexpressed in human CRC

**Figure 1:**
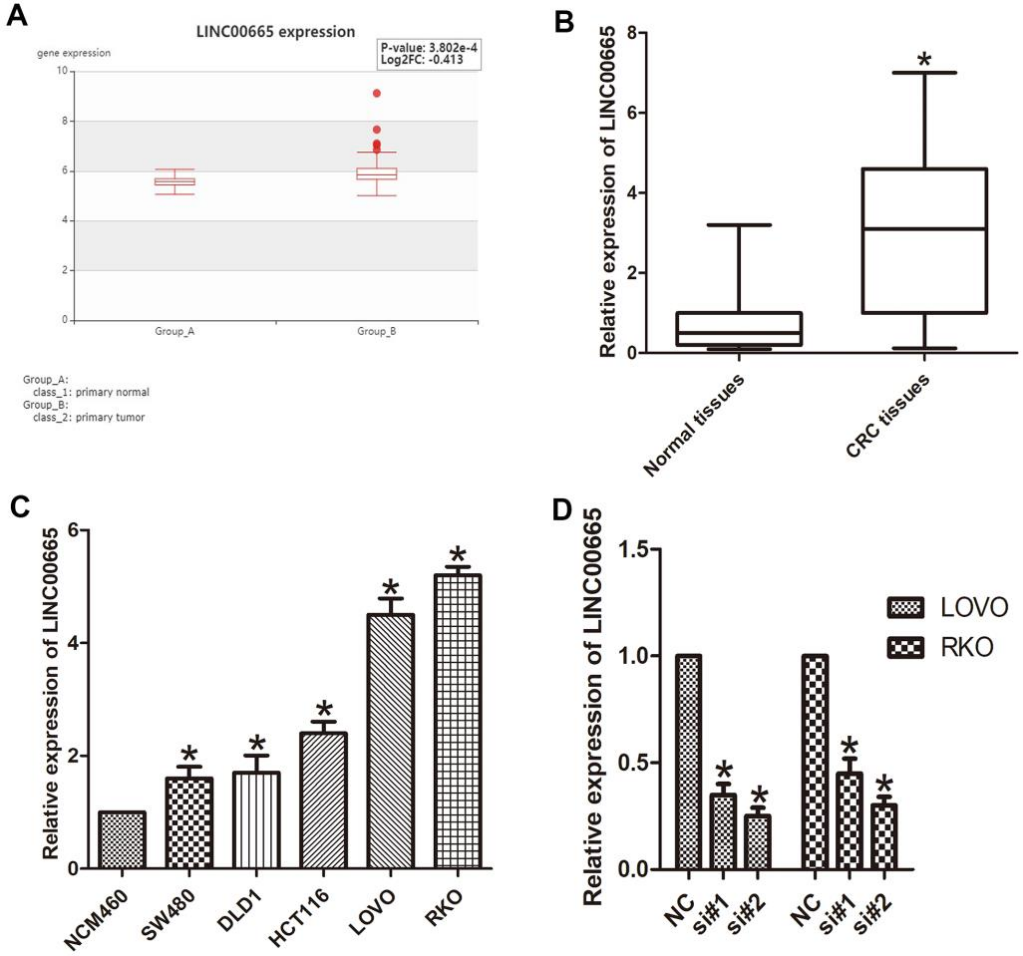
**LINC00665 expression was upregulated in CRC tissues and cell lines.** (**A**) LINC00665 was overexpressed in CRC tissues as indicated by HCMDB data analysis (*P < 0.01). (**B**) qPCR was used to measure the expression level of LINC00665 (*P < 0.01). (**C**) The expression levels of LINC00665 in multiple CRC cell lines compared with NCM460 normal colonic epithelial cells (n = 6; *P < 0.05 vs NCM460). (**D**) The knockdown efficiencies were examined in LOVO cells and RKO cells transfected with si-LINC00665 (si#1 and si#2; n = 6; *P < 0.05 vs NC).

To identify the role of LINC00665 in CRC, we analyzed the data obtained from a bioinformatics website (HCMDB data set), and the data showed that LINC00665 was significantly increased in CRC tissues (*P* < 0.05; Figure 1A). We then evaluated the levels of LINC00665 expression in CRC tumor tissues via qPCR, which showed significant upregulation of this lincRNA specifically in CRC samples (*P* < 0.05; Figure 1B). We also determined that LINC00665 expression in various CRC cell lines (LOVO, HCT116, DLD1, SW480, and RKO) was upregulated compared with that in the normal colonic cell line (NCM460, n = 6; *P* < 0.05; Figure 1C). The results from *in vitro* experiments indicated that the expression of LINC00665 in LOVO and RKO cells was even higher than that in other cells (n = 6; *P* < 0.01; Figure 1C), and these cells were chosen for further investigation. To determine the knockdown efficiencies in CRC cells, LINC00665-targeting siRNAs (si-LINC00665; si#1 and si#2) were used. We found two siRNAs (si#1 and si#2) with higher transfection efficiency, which were selected for further experiments (n = 6; *P* < 0.05; Figure 1D). Together, the above results indicate that LINC00665 may be associated with CRC aggressiveness.

### LINC00665 knockdown inhibited CRC cell proliferation

**Figure 2:**
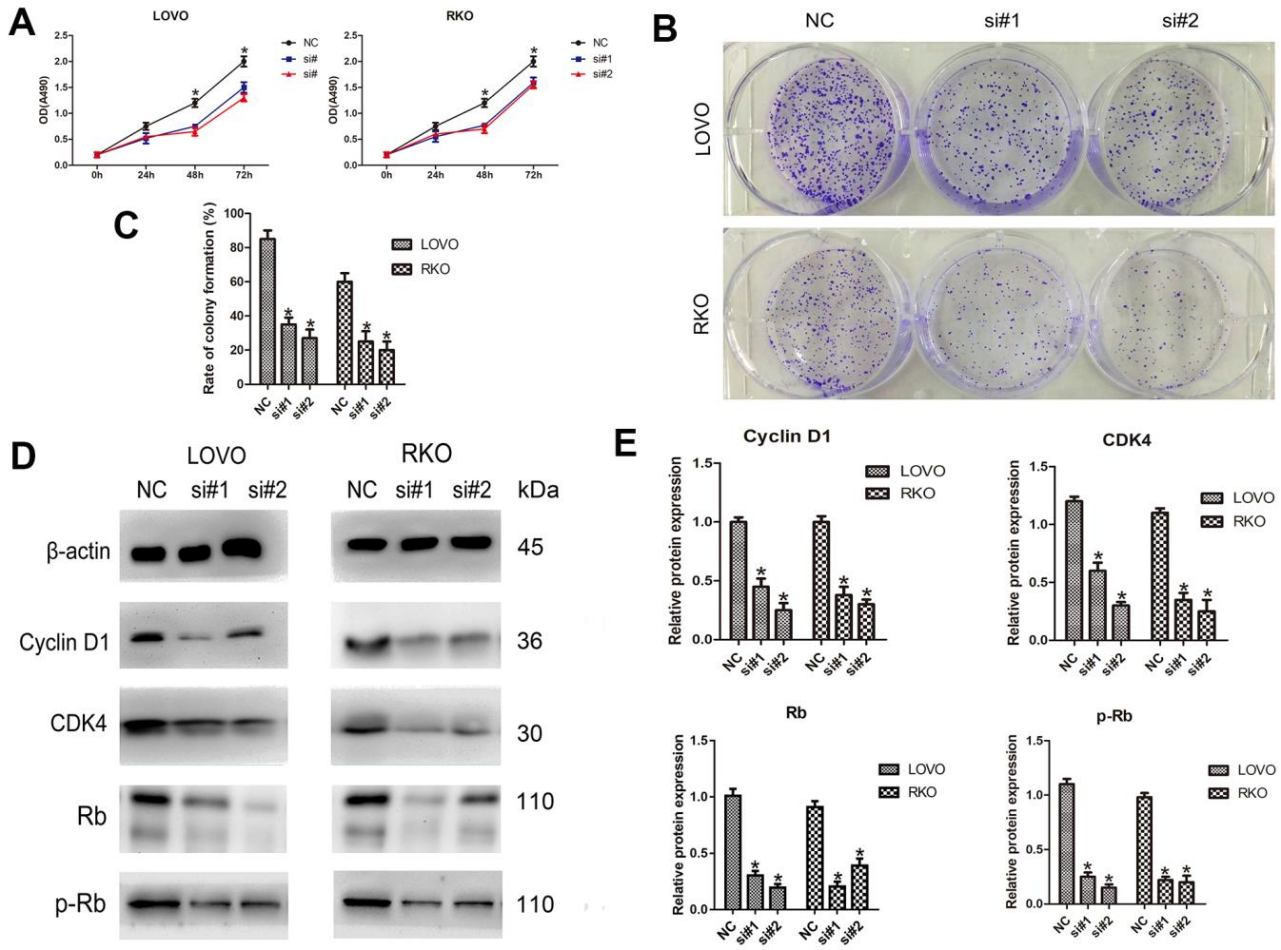
**LINC00665 knockdown inhibits the proliferation of CRC cells.** (**A**) The CellTiter 96® AQ_ueous_ One Solution Cell Proliferation assay showed different growth curves of LOVO and RKO cells after LINC00665 knockdown (n = 6; *P < 0.05 vs NC). (**B**, **C**) Colony formation analysis showed that the rates of colony formation in the LINC00665-knockdown groups were reduced compared with the control (NC) (n = 6; *P < 0.05 vs NC). (**D**, **E**) Cell cycle-related protein (cyclin D1, CDK4, Rb, and p-Rb) expression levels in the control (NC) and LINC00665-knockdown groups were determined by Western blotting (n = 6; *P < 0.05 vs NC).

To determine the effect of LINC00665 on cell proliferation, the CellTiter 96^®^ AQ_ueous_ One Solution Cell Proliferation assay was performed. We found that LINC00665 knockdown suppressed the proliferation of CRC cell lines (n = 6, *P* < 0.05; Figure 2A). In addition, colony formation assays showed that the colony formation number was significantly reduced after silencing LINC00665 in CRC cells (n = 6, *P* < 0.05; Figure 2B, 2C). To further investigate the underlying mechanism, several important cell cycle-related proteins (cyclin D1, cyclin-dependent kinase 4 (CDK4), Rb and phosphorylated Rb (p-Rb)) were investigated. The protein levels of cell cycle-related proteins were decreased after LINC00665 knockdown in CRC cells (n = 6, *P* < 0.05; Figure 2D, 2E). Taken together, our data demonstrated that knockdown of LINC00665 suppressed proliferation in CRC.

### LINC00665 knockdown promoted CRC cells apoptosis

**Figure 3:**
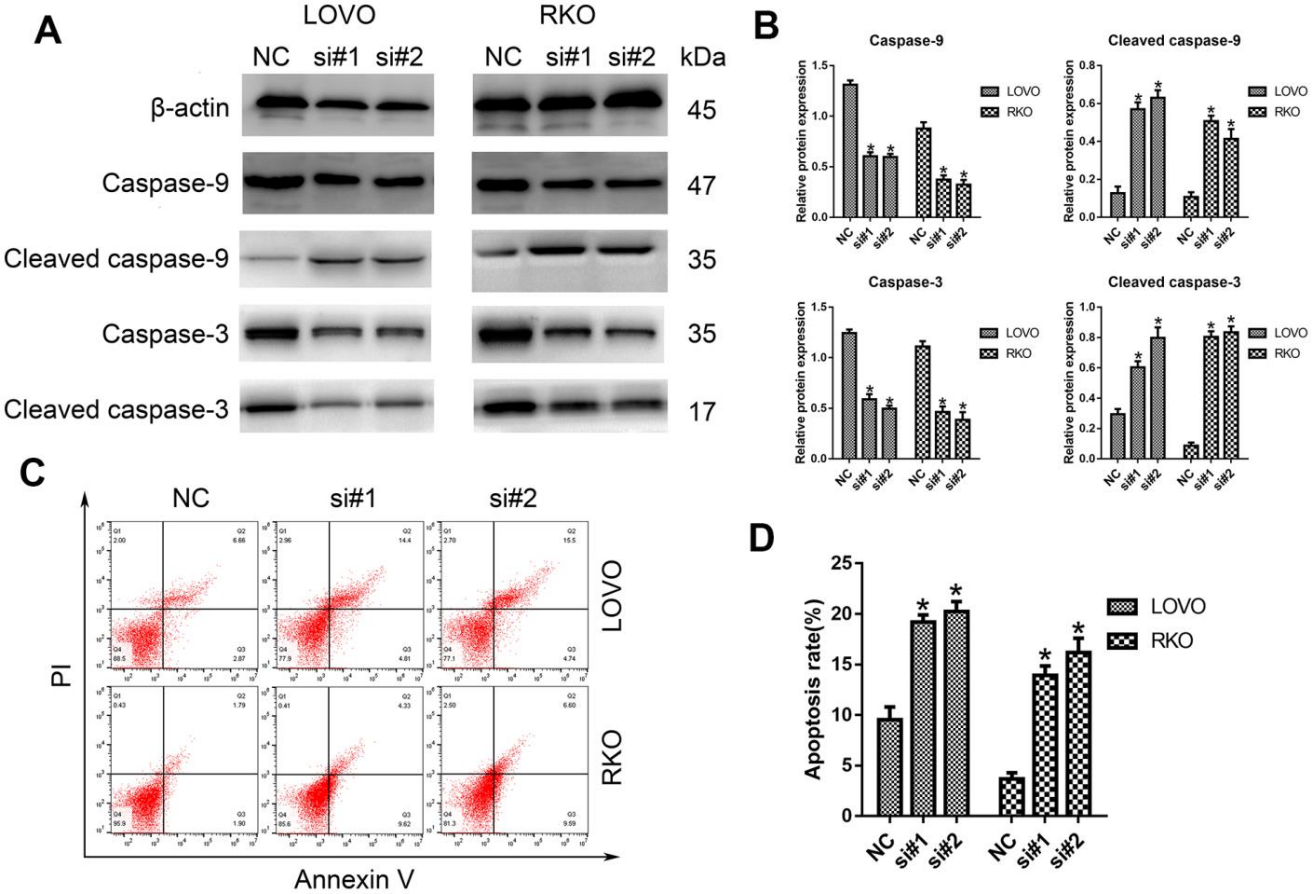
**Knockdown of LINC00665 induced apoptosis in CRC cells.** (**A**) Apoptosis-related protein levels were analyzed by Western blotting (n = 6; *P < 0.05 vs NC). (**B**) Western blotting showed the protein levels in both cell lines (n = 6; *P < 0.05 vs NC). (**C**, **D**) The apoptosis rates of LOVO and RKO cells after siRNA treatment were detected by flow cytometry.

Furthermore, the potential influence of LINC00665 on cell apoptosis was assessed by Western blotting analysis and flow cytometry. As shown in Figure 3A, 3B, we demonstrated that the protein levels of cleaved caspase-9 and caspase-3 were much higher after silencing LINC00665 than in the NC group. Similarly, the apoptosis rates of CRC cells were significantly elevated in the si-LINC00665 groups compared to the NC group (n = 6, *P* < 0.05; Figure 3C, 3D). These results indicate that the downregulation of LINC00665 induced apoptosis in CRC cells, suggesting that LINC00665 plays an oncogenic role.

### LINC00665 functioned as a ceRNA to sponge miR-126-5p

**Figure 4:**
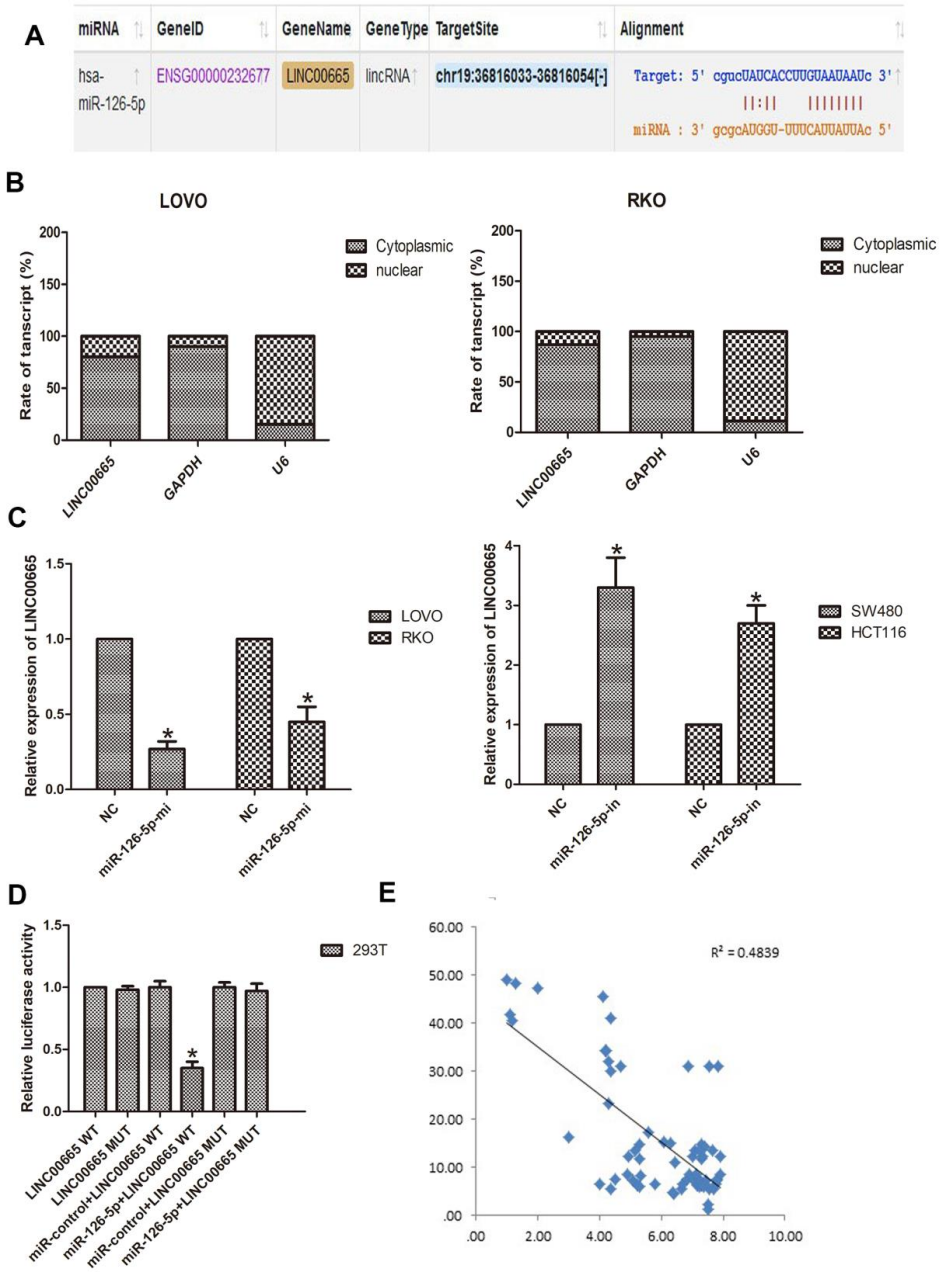
**LINC00665 is a direct target of miR-126-5p and is inversely related to miR-126-5p in CRC.** (**A**) Predicted miR-126-5p binding sites in the LINC00665 sequence. (**B**) Subcellular fractionation and qPCR analysis confirmed that LINC00665 was largely enriched in the cytoplasm of cells. (**C**) qPCR showed LINC00665 expression in cells transfected with a miR-126-5p inhibitor or mimic (n = 6, *P < 0.05 vs NC). (**D**) Luciferase reporter assay revealed the binding relationship between LINC00665 and miR-126-5p (n = 6, *P < 0.05 vs NC). (**E**) miR-126-5p mRNA levels were plotted against LINC00665 expression in CRC specimens, demonstrating a significant negative correlation (two-tailed Pearson’s correlation, r = 0.4839; *P* < 0.01).

To further investigate this hypothesis, we aimed to identify the miRNAs that might be targeted by LINC00665 using starBase tools (http://starbase.sysu.edu.cn). The predicted targets showed that miR-126-5p was a potential downstream target of LINC00665, and the potential binding site is presented (Figure 4A). To further reveal the underlying mechanism of LINC00665 in CRC malignant phenotypes, the subcellular distribution of LINC00665 was analyzed in CRC cancer cells. The results illustrated that LINC00665 was mostly distributed in the cytoplasm (n = 6, *P* < 0.05; Figure 4B) and further suggested that LINC00665 can function as a miRNA sponge and thus regulate target gene expression. Then, the regulatory relationship between LINC00665 and miR-126-5p was further investigated by qPCR. The results demonstrated that cell transfection with the miR-126-5p mimic reduced LINC00665 levels (n = 6, *P* < 0.05; Figure 4C); however, the miR-126-5p inhibitor upregulated LINC00665 expression (n = 6, *P* < 0.05; Figure 4C). To verify the role of LINC00665 as a target of miR-126-5p, we constructed LINC00665 luciferase reporters containing the WT (LINC00665-WT) and MUT (LINC00665-MUT) miR-126-5p binding sites. The dual-luciferase reporter assay showed that luciferase activity was reduced after transfection with the miR-126-5p mimic in the LINC00665-WT group; however, luciferase activity was not changed after cotransfection with the miR-126-5p mimic and LINC00665-mut (n = 6, *P* < 0.05; Figure 4D). Furthermore, Pearson’s correlation analysis revealed that LINC00665 and miR-126-5p were negatively correlated in CRC tissues (Figure 4E).

### MiR-126-5p is involved in LINC00665-mediated proliferation of CRC cells

**Figure 5:**
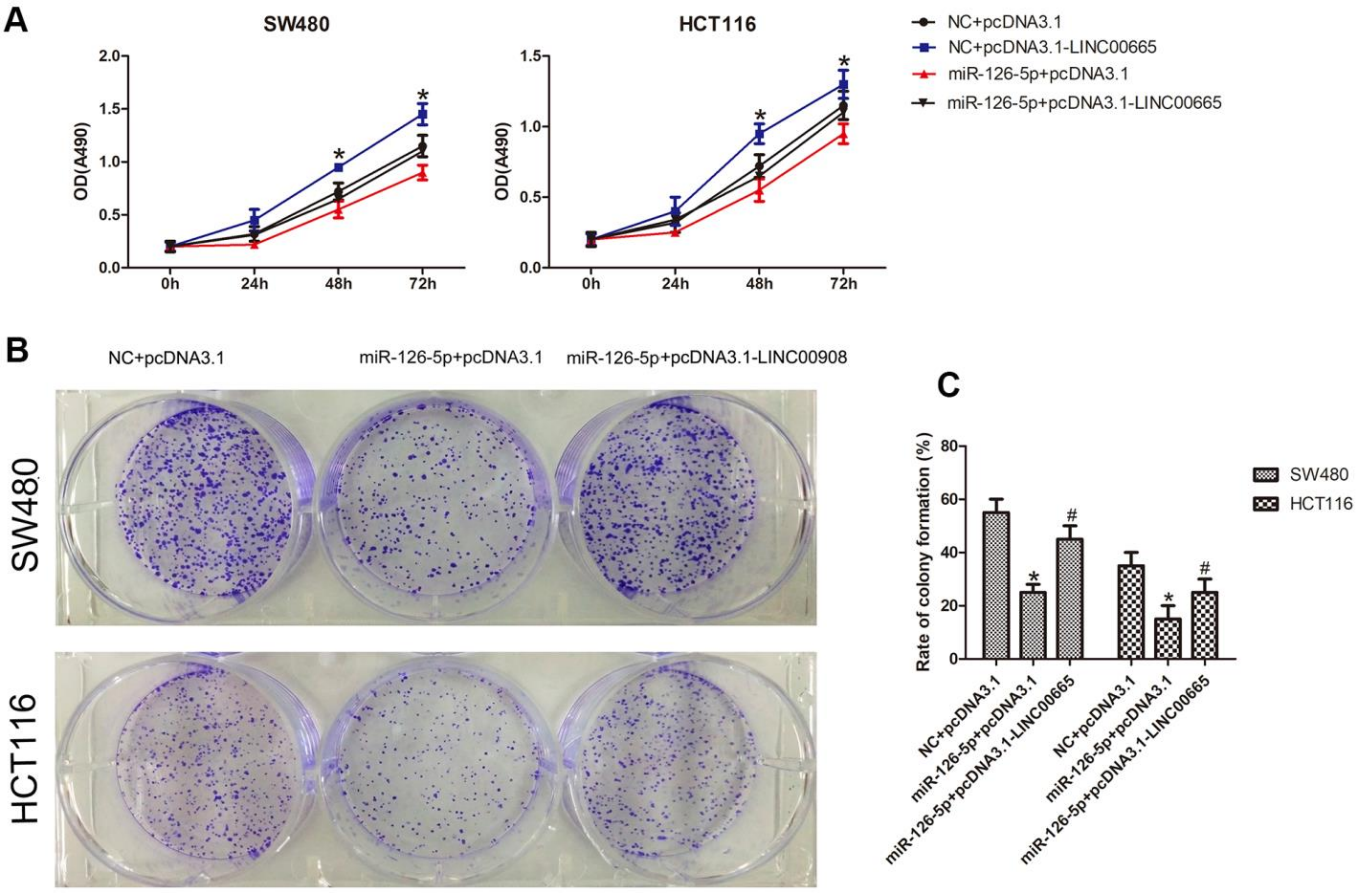
**MiR-126-5p inhibits LINC00665 function.** (**A**) CellTiter 96® AQ_ueous_ One Solution Cell Proliferation assays of cells cotransfected with an NC mimic or miR-126-5p mimic and a control plasmid (pcDNA3.1) or LINC00665 expression plasmid (pcDNA3.1-LINC00665) (n = 6; *P < 0.05 vs NC+pcDNA3.1; #*P* < 0.05 vs NC+pcDNA3.1-LINC00665). (**B**, **C**) Cells cotransfected with the NC mimic or miR-126-5p mimic and LINC00665 expression plasmid were subjected to a colony formation assay (n = 6; *P < 0.05 vs NC+pcDNA3.1; #*P* < 0.05 vs miR-126-5p +pcDNA3.1).

Next, to evaluate whether the biological effects associated with LINC00665 expression could be reversed by restoring miR-126-5p expression, we cotransfected the LINC00665 overexpression plasmid and miR-126-5p mimic into CRC cells. The Cell Titer 96® AQ_ueous_ One Solution Cell Proliferation assay demonstrated that miR-126-5p decreased the number of CRC cells, whereas cotransfection of the LINC00665 expression plasmid and miR-126-5p mimic showed that the LINC00665-induced increase in the number of CRC cells was ameliorated after miR-126-5p transfection (n = 6, *P* < 0.05; Figure 5A). As shown by colony formation assays, CRC colony formation was inhibited after transfection with miR-126-5p (n = 6, *P* < 0.05; Figure 5B, 5C). Accordingly, restoration of miR-126-5p expression ameliorated the LINC00665-induced increase in colony formation (n = 6, *P* < 0.05; Figure 5B, 5C). These results indicate that the impact of LINC00665 on cell proliferation was largely diminished upon miR-126-5p expression in CRC cells.

### LINC00665 sponges miR-126-5p to regulate PAK2 and FZD3

**Figure 6:**
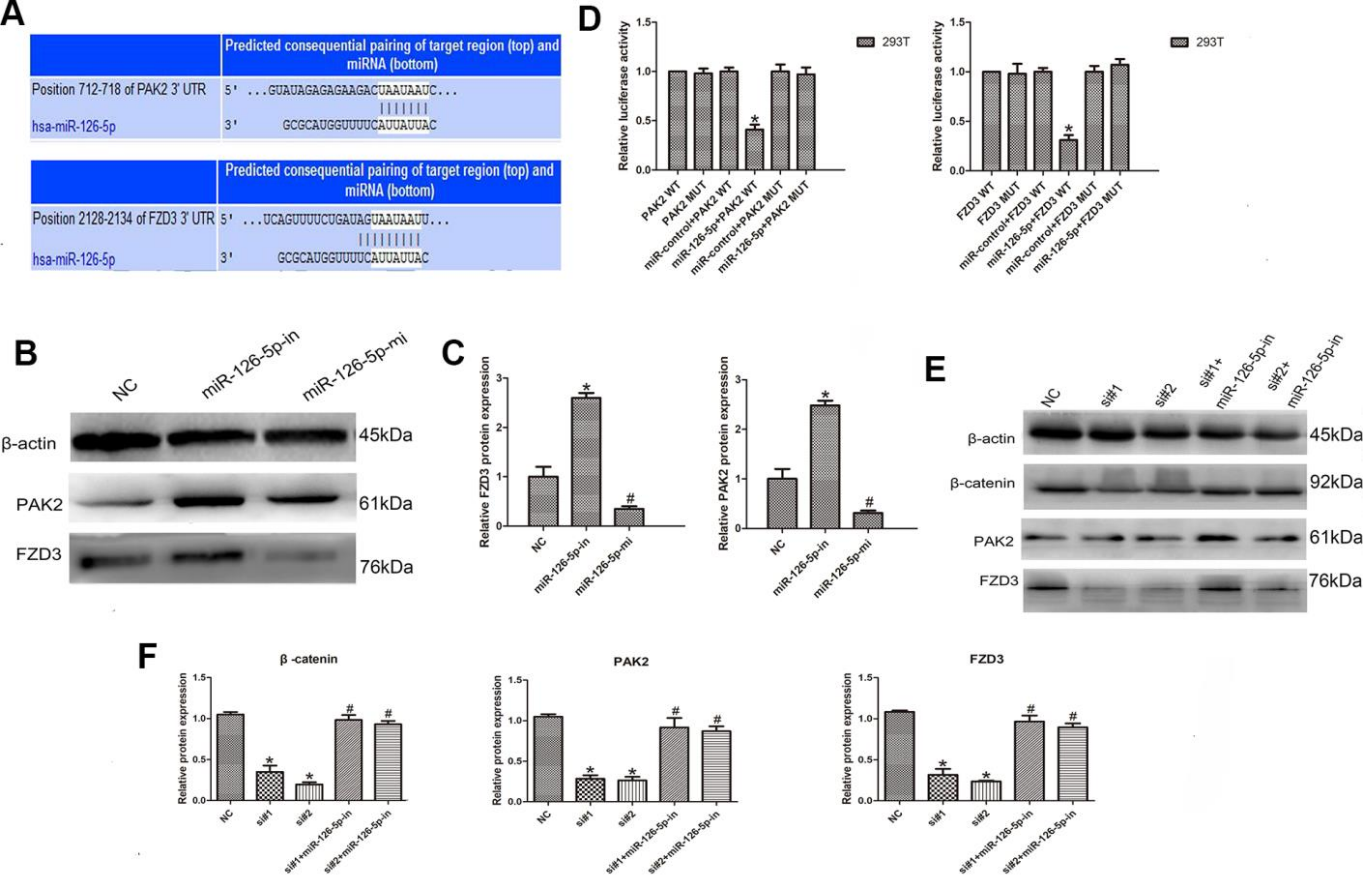
**LINC00665 regulates PAK2 and FZD3 expression by competitively binding with miR-126-5p in CRC.** (**A**) Predicted miR-126-5p binding sites in the PAK2 and FZD3 sequences. (**B**, **C**) Western blotting was used to detect the levels of PAK2 and FZD3 in cells transfected with the miR-126-5p mimic and inhibitor (n = 6; *P < 0.05 vs NC). (**D**) Luciferase assays in 293T cells transfected with miR-126-5p and wild-type or mutant PAK2 and the FZD3 target sequence (n = 6, *P < 0.05 vs NC). (**E**, **F**) Western blotting showed the expression of PAK2, FZD3 and β-catenin in cells transfected with si-LINC00665 or cotransfected with si-LINC00665 and miR-126-5p inhibitor (n = 6; *P < 0.05 vs NC; #*P* < 0.05 vs si-LINC00665).

To identify the miR-126-5p target gene, TargetScan database analysis showed that PAK2 and FZD3 were potential targets of miR-126-5p (Figure 6A). In addition, Western blot analyses detected a significant downregulation of PAK2 and FZD3 in cells after transfection with the miR-126-5p mimic and upregulation of PAK2 and FZD3 in cells after miR-126-5p inhibitor transfection (n = 6, *P* < 0.05; Figure 6B, 6C). Luciferase reporter assays further tested the interaction of these RNAs. As the data from the assays showed, the luciferase activity was significantly decreased after cotransfection of PAK2-WT or FZD3-WT and miR-126-5p mimics; however, the luciferase activity was not changed after transfection with PAK2-MUT or FZD3-MUT and miR-126-5p mimics (n = 6, *P* < 0.05; Figure 6D). Additionally, we transfected CRC cells with LINC00665-specific siRNAs with or without a miR-126-5p inhibitor and found that the decrease in PAK2 and FZD3 expression induced by LINC00665 knockdown could be rescued by inhibiting miR-126-5p (n = 6, *P* < 0.05; Figure 6E, 6F). Previous studies showed that tumorigenic FZD3 signaling was independent of the canonical WNT pathway [13]. We found that the decreased levels of β-catenin in cells with LINC00665 knockdown were partly restored after transfection with the miR-126-5p inhibitor (n = 6, *P* < 0.05; Figure 6E, 6F). In conclusion, LINC00665 regulates PAK2 and FZD3 expression by regulating miR-126-5p.

## DISCUSSION

The aberrant expression of lncRNAs plays key roles in the biological behavior of a variety of cancers. Currently, research on the regulatory roles and mechanisms of lncRNAs in cancer has become increasingly in-depth [21–23]. However, the underlying molecular mechanisms of lincRNAs in CRC remain unknown. Many of these molecules have been found to be dysregulated in cancer, but in-depth research reports are limited [24]. Accumulating studies have revealed the crucial role of LINC00665 in multiple cancers, including gastric cancer [25], breast cancer [26] and hepatocellular carcinoma [27]. However, few studies have explored the function and potential mechanism of LINC00665 in CRC. In this study, we revealed that LINC00665 expression was upregulated in CRC tissues and cells. Furthermore, to further uncover the role of LINC00665 in CRC, we detected the CRC cell lines’ biological behavior.

Therefore, we found through siRNA function experiments that the proliferation of CRC cells was significantly inhibited after LINC00665 downregulation. The cell cycle controls the proliferation of different cells through highly conserved and strictly regulated biological processes [28]. Studies have confirmed that the interaction between cyclin D1 and CDK4 accelerates the G1 phase transition of cells, which further increases Rb phosphorylation (p-Rb) [29, 30]. Herein, we further detected cell cycle-related protein expression in CRC cells. This study demonstrated that LINC00665 knockdown caused a reduction in the expression of cyclin D1, CDK4, and p-Rb. Caspase-3 and caspase-9 play an important role in the activation of downstream DNA cleavage molecules [31]. Activated caspases are an important way to induce apoptosis in cancer cells. We found that knockdown of LINC00665 activated caspase-9 and caspase-3 expression to further induce apoptosis. Overall, the focus of this study was to understand the role of LINC00665 in CRC, and the results suggest that LINC00665 might become a potential therapeutic target for CRC.

Accumulating evidence indicates that lncRNAs are used as ceRNAs of endogenous miRNAs to bind to miRNAs, and this function competitively inhibits the binding of miRNAs to their targets. A recent study showed that LINC00665 sponges miR-9-5p to further promote CRC progression by increasing ATF1 expression [31]. Similarly, we provide novel evidence of a ceRNA regulatory network in which LINC00665 functions as a miRNA sponge. We found that LINC00665 directly binds to miR-126-5p by bioinformatics analysis and dual-luciferase assays. Further evidence showed that miR-126-5p is negatively correlated with LINC00665. It has been demonstrated that miR-126-5p plays a role as a tumor suppressor in many cancers [32, 33], including CRC. Additionally, to confirm that LINC00665 competitively binds to miR-126-5p, related functional experiments were used. We demonstrated that LINC00665 could be a molecular sponge to directly offset the inhibitory action of miR-126-5p in CRC cells. Thus, the roles of LINC00665 in CRC cell-induced proliferation might occur through a candidate ceRNA of miR-126-5p.

In the present study, it was further determined that FZD3 and PAK2 were target genes of miR-126-5p through database screening, cell culture experiments, and dual luciferase reporter assays. FZD3 can increase the activation and stability of β-catenin protein by binding to the relevant ligands of the WNT pathway and further activate the transcription of downstream target genes [10]. We found that LINC00665 regulated β-catenin by interacting with endogenous miR-126-5p. Studies have found that PAK2 plays important roles in tumor cell proliferation, invasion, apoptosis and so on [34, 35]. Many studies have found that PAK2 is abnormally expressed in many tumors and can even be used as a tumor treatment target [36–38]. In this study, we found that knockdown of LINC00665 could inhibit FZD3 and PAK2 expression, while miR-126-5p inhibition abolished this phenomenon. In addition, LINC00665 caused the proliferation of CRC cells by sponging miR-126-5p to prevent miR-126-5p from inhibiting FZD3 and PAK2, which implied a potential mechanism for the roles of LINC00665 as an oncogene.

In summary, the above results showed that LINC00665 acts as an oncogenic lncRNA to facilitate the progression of CRC by sponging miR-126-5p and increasing FZD3 and PAK2 expression. This study mainly sheds light on the molecular mechanisms by which LINC00665 promotes CRC proliferation and inhibits cell apoptosis. This study may highlight a novel role of LINC00665 as a therapeutic target in future CRC therapy.

## MATERIALS AND METHODS

### Patients and tissue samples of CRC

Sixty-seven samples of CRC tissues and their corresponding adjacent nontumor tissues were provided by The Affiliated Hospital of Qingdao University. The samples were immediately collected, frozen and stored at −80° C until use. Informed consent forms were obtained from each patient, and the present study was approved by the Hospital Ethics Review Committee.

### Cell lines and medium

The human CRC cell lines DLD1, RKO, HCT116, LOVO, SW480 and NCM460 were obtained from the Institute of Biochemistry and Cell Biology of the Chinese Academy of Sciences (Shanghai, China). The cells were grown in RPMI 1640 (Invitrogen, CA, USA) with 10% fetal bovine serum (Thermo Fisher Scientific, Waltham, MA, USA), 100 U/ml penicillin, and 100 mg/ml streptomycin (Gibco, Grand Island, NY) and incubated at 37° C with 5% CO2 in a humidified atmosphere.

### RNA extraction and real-time quantitative polymerase chain reaction (qPCR)

TRIzol reagent (Invitrogen, Carlsbad, CA, USA) was used to extract total RNA from CRC tissues and cells. cDNA was synthesized and generated by the PrimeScript RT Reagent Kit (TaKaRa, Dalian, China). A SYBR Green PCR Master mix kit (TaKaRa, Dalian, China) was used following the manufacturer’s instructions. GAPDH was used as the internal control, GAPDH, and the 2^−ΔΔCt^ method was used to calculate relative fold change expression. The primers were provided by GenePharma (Shanghai, China). The sequences of all primers used in the experiment were as follows: LINC00665, forward: 5′-GGTGCAAAGTGGGAAGTGTG-3′ and reverse: 5′-CGGTGGACGGATGAGAAACG-3′; GAPDH, forward: 5′-GGGAGCCAAAAGGGTCATCA-3′ and reverse: 5′-TGATGGCATGGACTGTGGTC-3′.

### Bioinformatic analyses

The putative target genes of LINC00665 and miR-126-5p were predicted by DIANA-LncBase Predicted v.2 (http://carolina.imis.athena-innovation.gr/diana_toolsd), starBase (http://starbase.sysu.edu.cn/) and TargetScan 7.2 (http://www.targetscan.org/).

### Cell transfection

LINC00665 siRNAs (si#1 and si#2), miR-126-5p mimics, negative control (NC) mimic, miR-126-5p inhibitors, NC inhibitor, LINC00665 expression vector and NC plasmid were obtained from GenePharma (Shanghai, China). and the sequences are shown in the Supplementary Data. Lipofectamine 3000 reagent (Life Technologies, Grand Island, NY, USA) was used to transfect the above cells.

### Dual luciferase reporter assay

The pcDNA3.1(+) expression vector (Life Technologies, Grand Island, NY, USA) was used to clone LINC00665 fragments containing the wild-type (WT) or mutated (MUT) miRNA target sequence. FZD3 and PAK2 fragments containing the WT or MUT target sequence were provided by GenePharma (Shanghai, China). The Dual Luciferase Reporter Assay System (Promega, Madison, WI, USA) was used to quantify the luciferase activities. All procedures followed the manufacturer’s protocol.

### Cell proliferation assay

All cells were plated in 96-well plates at 37° C. After culturing for 24 h, the cells were transfected with siRNA. Then, a Cell Counting Kit (CellTiter 96^®^ AQ_ueous_ One Solution Cell Proliferation Assay kit, Promega, USA) was added, and each cell well was tested at each time point (0 h, 24 h, 48 h, and 72 h). A SpectraMax M5 multifunctional microplate reader (Molecular Devices, Sunnyvale, CA, USA) was used to measure the optical density at 490 nm.

### Colony formation assay

Approximately 500 siRNA-transfected cells were seeded on a six-well plate for 24 hours, cultured for 7 days, washed with PBS, and fixed with 4% paraformaldehyde for approximately 30 minutes. Then, images were captured by a digital camera (Sony, Tokyo, Japan). ImageJ software was used to counter the colonies. The rate of colony formation was calculated as the number of colonies formed by inoculated cells multiplied by 100%.

### Subcellular fractionation

Cytoplasmic and nuclear RNA from CRC cells was extracted by a nuclear and cytoplasmic protein extraction kit (Beyotime, Shanghai, China) according to the manufacturer’s protocol. The cytoplasmic and nuclear RNA expression ratios were determined by qPCR. The cytoplasmic and nuclear controls were GAPDH and U6, respectively.

### Flow cytometric assay

After culturing the siRNA-transfected cells for 48 hours, flow cytometry analysis was performed. Fluorescein isothiocyanate-Annexin V and propidium iodide (Absin, Shanghai, China) were used to stain 300 μl binding buffer of cell suspension. Apogee Flow Cytometers (Apogee Flow Systems, Hemel Hempstead, UK) were used to analyze the stained cells.

### Western blotting assay

RIPA buffer (Thermo Fisher Scientific, Waltham, Massachusetts, USA) was used to extract the protein from CRC tissue and cells for Western blotting assay. The protein was separated using 10% SDS-PAGE, and then a polyvinylidene fluoride (PVDF) membrane was used to transfer protein followed by blocking with 5% nonfat milk. The primary antibodies were as follows: anti-cyclin D1, CDK4, Rb, p-Rb (Ser807/811), procaspase-9, cleaved caspase-9, procaspase-3, cleaved caspase-3, PAK2, β-catenin, β-actin (1:1000, Cell Signaling Technology, Beverly, MA, USA) and anti-FZD3 (1:1000, Abcam, Cambridge, MA, USA). Then, the membrane was incubation at 4° C for 12 h. After three washes, the membrane was incubated with HRP-labeled secondary antibody (Cell Signaling Technology, USA), incubated for 1 h at room temperature, developed with an enhanced chemiluminescence kit (Millipore, Billerica, MA, USA) and captured by X-ray film.

### Statistical analysis

Student’s *t*-test was used to analyze the differences between two groups. Differences between more than two groups were analyzed using one-way analysis of variance (ANOVA) followed by a post hoc test. The data were calculated as standard deviations (SDs). A value of less than 0.05 was considered significant.

## Supplementary Material

Supplementary Data
