# Supplementary material for: Long intergenic noncoding RNA 00665 promotes proliferation and inhibits apoptosis in colorectal cancer by regulating miR-126-5p
Source: Aging (Albany NY). 2021 Apr 20;13(10):13571–84. doi: 10.18632/aging.202874 (PMC8202867; doi:10.18632/aging.202874)
Supplement: Supplementary Data [file aging-13-202874-s001.pdf]

SUPPLEMENTARY DATA

Supplementary Table 1. All siRNA sequences used in this study.

Name	Sequence
NC/ NC mimic-F	UUCUCCGAACGUGUCACGUTT
NC/NC mimic-R	ACGUGACACGUUCGGAGAATT
LINC00665 si#1-F	GCUGAAUAGUUCUGUAUGUTT
LINC00665 si#1-R	ACAUACAGAACUAUUCAGCTT
LINC00665 si#2-F	CCACCUUUCUUGUGGUUCUTT
LINC00665 si#2-R	AGAACCACAAGAAAGGUGGTT
miR-126-5p mimic-F	CAUUAUUACUUUUGGUACGCG
miR-126-5p mimic-R	CGUACCAAAAGUAAUAAUGUU
NC inhibitor	CAGUACUUUUGUGUAGUACAA
miR-126-5p inhibitor	CGCGUACCAAAAGUAAUAAUG

Sequence of the putative miR-126-5p binding site in luciferase reporters

LINC00665-WT:
AGAATGCAATTAACTGATAAGATACTGTGGCA
AGCTATATCCGCAGTTCCCAGGAATTCGTCCAA
TTGATTACACCCAAAAGCCCCGCGTCTATCACC
TTGTAATAATCTTAAAGCCCCTGCACCTGGAAC
TATTAACGTTCCTGTAACCATTTATCCTTTTAAC
TTTTTTGCCTACTTTATTTCTGTAAAATTGTTTT
AACTAGACC

LINC00665-MUT:
AGAATGCAATTAACTGATAAGATACTGTGGCA
AGCTATATCCGCAGTTCCCAGGAATTCGTCCAA
TTGATTACACCCAAAAGCCCCGCGTCTATCACC
TTTGCCGCCGCTTAAAGCCCCTGCACCTGGAAC
TATTAACGTTCCTGTAACCATTTATCCTTTTAAC
TTTTTTGCCTACTTTATTTCTGTAAAATTGTTTT
AACTAGACC

The LINC00665 sequence in overexpression plasmid (pcDNA3.1 vector)

LINC00665 2856bp
ENST00000591372.2
CCTCCCATTTCCGGAAGCCTGGCGCAAGGTTGGT
CTGCAGGTGGCCTCCAGGTGCAAAGTGGGAAGT
GTGAGTCCTCAGTCTTGGGCTATTCGGCCACGTG
CCTGCCGGACATGGGACGCTGGAGGGTCAGCAG
CGTGGAGTCCTGGCCTTTTGCGTCCACGGGTGGG
AAATTGGCCATTGCCACGGCGGGAACTGGGACT
CAGGCTGCCCCCCGGCCGTTTCTCATCCGTCCAC
CGGACTCGTGGGCGCTCGCACTGGCGCTGATGT
AGTTTCCTGACCTCTGACCCGTATTGTCTCCAGA
TTAAAGGTACGACATTTGGAGGCCCCAGCGAGA
AACGTCACCGGGAGAAACGTCACCGGGCGAGA
GCCGGGCCCGCTGTGTGCTCCCCCGGAAGGACA
GCCAGCTTGTAGGGGGGAGTGCCACCTGAAAAA
AAAATTTCCAGGTCCCCAAAGGGTGACCGTCTTC
CGGAGGACAGCGGATCGACTACCATGTGGGTGC
CCACAAAAATTCCACCTCTGAGTCCTCAACTGCT
GACCCCGGGGTCAGGAACTGTTGGAAACTACTG
AAACTGGCCGACCTGATCTTCAAAATGTGCCCCT
AGGAAAGGTGGATGCCACCGTGTTCACAGACAG
TAGCAGCTTCCTCGAGAAGGGACTACGAAAGGC
CGGTGCAGCTGTTACCATGGAGACAGATGTGTT
GTGGGCTCAGGCTTTACCAGCAAACACCTCAGC
ACAAAAGGCTGAATTGATCGCCCTCACTCAGGC
TCTCCGATGGGGTAAGGATATTAACGTTAACACT
GACAGCAGGTACGCCTTTGCTACTGTGCATGTAC
GTGGAGCCATCTACCAGGAGCGTGGGCTACTCA
CCTCAGCAGGTGGCTGTAATCCACTGTAAAGGA
CATCAAAAGGAAAACACGGCTGTTGCCCGTGGT
AACCAGAAAGCTGATTCAGCAGCTCAAGATGCA
GTGTGACTTTCAGTCACGCCTCTAAACTTGCTGC
CCACAGTCTCCTTTCCACAGCCAGATCTGCCTGA
CAATCCCGCATACTCAACAGAAGAAGAAAACTG
GCCTCAGAACTCAGAGCCAATAAAAATCAGGAA
GGTTGGTGGATTCTTCCTGACTCTAGAATCTTCA
TACCCCGAACTCTTGGGAAAACTTTAATCAGTCA
CCTACAGTCTACCACCCATTTAGGAGGAGCAAA
GCTACCTCAGCTCCTCCGGAGCCGTTTTAAGATC
CCCCATCTTCAAAGCCTAACAGATCAAGCAGCT
CTCCGGTGCACAACCTGCGCCCAGGTAAATGCC
AAAAAAGGTCCTAAACCCAGCCCAGGCCACCGT
CTCCAAGAAAACTCACCAGGAGAAAAGTGGGA
AATTGACTTTACAGAAGTAAAACCACACCGGGC
TGGGTACAAATACCTTCTAGTACTGGTAGACACC
TTCTCTGGATGGACTGAAGCATTTGCTACCAAAA
ACGAAACTGTCAATATGGTAGTTAAGTTTTTACT
CAATGAAATCATCCCTCGACATGGGCTGCCTGTT
GCCATAGGGTCTGATAATGGACCGGCCTTCGCCT
TGTCTATAGTTTAGTCAGTCAGTAAGGCGTTAAA
CATTCAATGGAAGCTCCATTGTGCCTATCGACCC
CAGAGCTCTGGGCAAGTAGAACGCATGAACTGC

ACCCTAAAAAACACTCTTACAAAATTAATCTTAG
AAACCGGTGTAAATTGTGTAAGTCTCCTTCCTTT
AGCCCTACTTAGAGTAAGGTGCACCCCTTACTGG
GCTGGGTTCTTACCTTTTGAAATCATGTATGGGA
GGGCGCTGCCTATCTTGCCTAAGCTAAGAGATG
CCCAATTGGCAAAAATATCACAAACTAATTTATT
ACAGTACCTACAGTCTCCCCAACAGGTACAAGA
TATCATCCTGCCACTTGTTCGAGGAACCCATCCC
AATCCAATTCCTGAACAGACAGGGCCCTGCCAT
TCATTCCCGCCAGGTGACCTGTTGTTTGTTAAAA
AGTTCCAGAGAGAAGGACTCCCTCCTGCTTGGA
AGAGACCTCACACCGTCATCACGATGCCAACGG
CTCTGAAGGTGGATGGCATTCCTGCGTGGATTCA
TCACTCCCGCATCAAAAAGGCCAACGGAGCCCA
ACTAGAAACATGGGTCCCCAGGGCTGGGTCAGG
CCCCTTAAAACTGCACCTAAGTTGGGTGAAGCC
ATTAGATTAATTCTTTTTCTTAATTTTGTAAAACA
ATGCATAGCTTCTGTCAAACTTATGTATCTTAAG
ACTCAATATAACCCCCTTGTTATAACTGAGGAAT
CAATGATTTGATTCCCCAAAAACACAAGTGGGG
AATGTAGTGTCCAACCTGGTTTTTACTAACCCTG
TTTTTAGACTCTCCCTTTCCTTTAATCACTCAGCC
TTGTTTCCACCTGAATTGACTCTCCCTTAGCTAA
GAGCGCCAGATGGACTCCATCTTGGCTCTTTCAC
TGGCAGCCGCTTCCTCAAGGACTTAACTTGTGCA
AGCTGACTCCCAGCACATCCAAGAATGCAATTA
ACTGATAAGATACTGTGGCAAGCTATATCCGCA
GTTCCCAGGAATTCGTCCAATTGATTACACCCAA
AAGCCCCGCGTCTATCACCTTGTAATAATCTTAA
AGCCCCTGCACCTGGAACTATTAACGTTCCTGTA
ACCATTTATCCTTTTAACTTTTTTGCCTACTTTAT
TTCTGTAAAATTGTTTTAACTAGACCCCCCCTCT
CCTTTCTAAACCAAAGTATAAAAGCAAATCTAG
CCCCTTCTTCAGGCCGAGAGAATTTCGAGCGTTA
GCCGTCTCTTGGCCACCAGCTAAATAAACGGATT
CTTCA
